# Social motivation and sensory over-responsivity: Independent correlates of picky eating severity in early childhood

**DOI:** 10.1016/j.appde.2026.100010

**Published:** 2026-09-10

**Authors:** Annamarie Nocera, Serena J. Gu, Rebecca F. Schwarzlose, Rebecca Tillman, Kirsten E. Gilbert

**Affiliations:** a Department of Psychiatry, Washington University in St. Louis, 4444 Forest Park Ave, Suite 2100, St. Louis, MO, 63108, USA; b Department of Psychological & Brain Sciences, Washington University in St. Louis, 1 Brookings Drive, St. Louis, MO, 63130, USA

**Keywords:** Picky eating, Early childhood, ARFID, Autistic traits, Social motivation, Sensory processing, Overcontrol

## Abstract

**Objective:**

Picky eating is common in children, but when persistent, is predictive of Avoidant/Restrictive Food Intake Disorder (ARFID). Multiple factors have been individually associated with picky eating severity in early childhood, but these rarely have been modeled simultaneously, leaving the relative importance of each unclear. Rather than categorical ARFID diagnosis, this exploratory study used a dimensional measure of picky eating severity in early childhood, identifying independent psychological and clinical correlates of picky eating severity at ages when picky eating first becomes clinically relevant.

**Method:**

323 community children aged 4–7 years (*M*(*SD*) = 5.96(1.10)) were oversampled for rigid, perfectionistic, and overcontrolled traits. Although autism spectrum disorder (ASD) was exclusionary, subclinical autistic traits were assessed. Candidate psychiatric symptoms and psychological constructs, including depression, anxiety, obsessive-compulsive disorder (OCD), attention-deficit/hyperactivity disorder (ADHD), autistic traits, sensory processing, disgust sensitivity, and overcontrol, were examined in linear regressions in relation to concurrent picky eating via the Nine Item ARFID Screen (NIAS-PE).

**Results:**

Individual linear regression models covarying for age demonstrated symptoms of depression, OCD, subclinical autistic traits, sensory over-responsivity, and overcontrolled temperament were each associated with picky eating severity. In a unified multivariable model, subclinical autistic traits (related primarily to social motivation deficits) and sensory over-responsivity emerged as the only independent correlates significantly associated with picky eating severity.

**Discussion:**

Subclinical ASD traits (particularly social motivation deficits) and sensory overresponsivity were independent correlates of dimensional picky eating severity at developmental ages when picky eating becomes non-normative. Findings should inform future work on ARFID risk stratification and treatment development.

## 1. Introduction

Picky eating, also sometimes referred to as selective eating, is a common feeding behavior in preschoolers. While definitions vary, picky eating is characterized by strong food preferences and an unwillingness to try new foods (Taylor et al., 2015). It is estimated around 20–30% of children experience picky eating (Bourne et al., 2023; Cole et al., 2017). Although even normative picky eating results in increased family mealtime stress and reduced nutrient intake (Trofholz et al., 2017; Taylor & Emmett, 2019), persistent or severe picky eating is associated with internalizing psychopathology and may predict later eating patterns (Zucker et al., 2015; Herle et al., 2019). Picky eating tends to peak in preschool and usually resolves by age 6 (Cardona Cano et al., 2015). Thus, improved understanding of the psychological context in which picky eating occurs during the first years in which it is non-normative is imperative for early risk identification and intervention.

Here, “picky eating” refers to dimensional eating behavior rather than a categorical Avoidant/Restrictive Food Intake Disorder (ARFID) diagnosis, though persistent picky eating is associated with elevated risk for ARFID (Breiner et al., 2024). ARFID is characterized by an eating or feeding disturbance — such as apparent lack of interest in food, avoidance based on sensory characteristics, or concern about aversive consequences of eating — that results in significant nutritional deficiency, weight loss, dependence on nutritional supplementation, or marked interference with psychosocial functioning (APA, 2013). Picky eating maps most closely onto the sensory sensitivity presentation of ARFID, which is characterized by food avoidance based on sensory qualities and limited dietary variety (Zickgraf & Ellis, 2018).

Various clinical presentations have been associated with picky eating. For example, depression and anxiety symptoms have both been concurrently and prospectively associated with picky eating in preschool-aged children (Zucker et al., 2015). Obsessive-compulsive symptoms have also been independently linked to childhood picky eating (Schwarzlose et al., 2022). The relationship between picky eating and ADHD is less clear, as some studies report associations between inattention symptoms and selective eating (Zucker et al., 2015; Kaşak et al., 2025), while others suggest ADHD is more closely associated with emotional overeating than restrictive patterns (Görgü, 2020). Additionally, picky eating is extremely common in autism spectrum disorder (ASD), with significantly higher rates than neurotypical peers (Baraskewich et al., 2021; Ferrara et al., 2025).

In addition to multiple psychiatric symptoms, various psychological processes (including cognitive, sensory and temperamental constructs) that are not tied to a specific diagnosis have also been implicated in picky eating. Sensory processing issues have a well-established link to picky eating in community samples (Campos-Sánchez et al., 2024). Additionally, the high rate of picky eating among autistic individuals is thought to be related to oral sensory sensitivities (Rodrigues et al., 2023). Beyond autism-specific pathways, another construct engendering risk for picky eating includes disgust sensitivity, which has been proposed as a proximal food-specific emotional response that reinforces and maintains food avoidance both in picky eating and in ARFID (Harris et al., 2019). Finally, overcontrol, a transdiagnostic personality style characterized by perfectionism, cognitive inflexibility/rigidity, and excessive need for control, has been implicated in restrictive eating disorders (Lynch, 2018; Isaksson et al., 2021). Overcontrol could theoretically map onto picky eating through multiple potential pathways: perfectionistic tendencies could show up as strict rules surrounding food acceptability, while excessive need for control and inflexibility may make the inherently unpredictable experience of trying new foods particularly difficult. Cognitive rigidity, a facet of overcontrol, is associated with picky eating (Gilbert, Perino, et al., 2020; Zickgraf et al., 2022), and overcontrol is evident in internalizing psychopathologies (OCD, anxiety, depression) associated with severe or persistent picky eating (Gilbert, Perino, et al., 2020; Lynch et al., 2015; Steinhoff et al., 2025). Despite the overlap, to our knowledge, overcontrol has not been directly studied in relation to picky eating. Psychological processes may underlie associations with picky eating and transdiagnostic clinical presentations, yet these candidate psychological risk factors are rarely examined in conjunction with co-occurring psychiatric symptoms, leaving the relative and independent contributions to picky eating severity unclear. Moreover, identifying psychological factors and co-occurring psychiatric symptoms associated with picky eating when picky eating is first emerging as non-normative is vital in understanding what factors may contribute to persistent and clinically meaningful picky eating behaviors that confer risk for ARFID.

This study addresses these gaps by examining concurrent transdiagnostic psychiatric clinical symptoms and psychological processes in relation to picky eating—assessed via the picky eating subscale score of the broader nine item ARFID screening measure (Zickgraf & Ellis, 2018)—in a community sample of children aged 4 to 7 years. Children were recruited to exhibit elevated overcontrolled temperamental traits (traits such as rigidity, inflexibility, and need for control) which have theoretical overlap with picky eating, while the 4–7 year age range provides a unique opportunity to examine the developmental period when picky eating becomes non-normative. The present study is exploratory and hypothesis-generating and seeks to clarify which psychological constructs independently relate to concurrent picky eating severity. To do this, we first examined candidate transdiagnostic clinical symptoms and psychological processes individually with picky eating, including depression, anxiety, OCD, and ADHD, autistic traits, disgust sensitivity, sensory processing, and overcontrolled temperament. We then examined candidate clinical and psychological processes simultaneously in relation to picky eating, making no specific hypotheses regarding results.

## 2. Methods

### 2.1. Participants

Participants were from the baseline assessment of the Neuro-development of Overcontrol and Anxiety (NOA) study, a community cohort of 4–7 year old children (and their caregivers) recruited from the St. Louis metropolitan area. Importantly, the NOA study oversampled for overcontrolled traits, including advertisements that sought out children with “perfectionistic, shy and/or self-critical tendencies,” to ensure that at least two-thirds of participants were elevated on overcontrolled temperament. Participants were recruited from July 2022 through July 2025. Recruitment methods included online and in-person flyering, research registries, community advertisements at local schools and child anxiety clinics, as well as social media postings in parent groups and social media postings via BuildClinical. Further detailed recruitment procedures and participants are described in the second study of Jennings et al. (2026).

Exclusion criteria included ASD diagnosis, neurologic disorders, severe developmental delays, serious medical conditions or severe head injuries, baseline psychotropic medication use, and inability to speak English. As part of the larger study, eligible parent-child dyads completed multiple assessments, including a child EEG, parent-child interaction tasks, and behavioral tasks at baseline and are currently completing two yearly follow-up sessions. Pertinent for the current study, children and caregivers completed a series of questionnaires to assess the child’s psychiatric symptoms, temperament, and feeding behaviors. Trained research staff conducted structured diagnostic interviews with parents using the Kiddie Schedule for Affective Disorders and Schizophrenia – Early Childhood (KSADS-EC; Gaffrey & Luby, 2012). Psychiatric diagnoses of the study population, determined using KSADS-EC, can be found in Table 1. Given the unique developmental trajectory, children meeting criteria for ASD were excluded from the sample, while dimensional autistic traits were retained via the Social Responsiveness Scale (SRS; Constantino et al., 2003). This design allows evaluation of the relationship between picky eating and autism-related traits independent of categorical diagnosis, which may be more relevant to the broader population. The final sample for the present study included 323 children (mean [SD] age = 5.96 [1.10] years old, 51.1% female). The study was approved by the Washington University in St. Louis Institutional Review Board and conformed to the Declaration of Helsinki standards. Written informed consent was obtained from a legal guardian for each child, and child verbal assent was obtained as developmentally appropriate prior to study participation. The study was not pre-registered.

### 2.2. Measures

#### Picky Eating.

The Nine Item ARFID Screen (NIAS; Zickgraf & Ellis, 2018) is a nine-item, parent-report screener to evaluate ARFID-related symptoms with three subscales: (1) picky eating (NIAS-PE; three items indicating selective eating based on sensory qualities of foods), (2) appetite/lack of interest (NIAS-A; three items), and (3) fear of aversive consequences (NIAS-F; three items). Items are rated on a six-point Likert scale from 0 (“Strongly Disagree”) to 5 (“Strongly Agree”). Each subscale score is calculated by summing items and ranges from 0 to 15. The current study uses the picky eating subscale (NIAS-PE) score as the primary outcome, with scores >9 indicating a positive screen for ARFID (Murray et al., 2021). In our sample, NIAS-PE scores had excellent internal consistency (α = .90), as well as NIAS-F (α = .95) and NIAS-A (α = .90), which were included in the supplement.

#### Depression and Anxiety Symptoms.

The Revised Children’s Anxiety and Depression Scale - Parent Version (RCADS; Chorpita et al., 2005) was used to assess child depression and anxiety symptoms. It is a 47-item parent-report questionnaire with items rated from 0 (“Never”) to 3 (“Always”). This scale computes T-scores that are standardized to age and sex. In the current study, the depression T-score and total anxiety T-score were included as separate predictors, both with moderate internal consistency in our sample (depression α = .66; anxiety α = .73).

#### Obsessive-Compulsive Symptoms.

The parent version of the Revised Children’s Obsessional Compulsive Inventory (ChOCI-R-P; Uher et al., 2008) was used to assess obsessive-compulsive symptom severity. It is a 38-item parent-report measure with items rated from 0 (‘Not at all’) to 2 (‘A lot’). The ChoOCI-R-P computes a total symptom score by summing the reported compulsion and obsession symptom scores, and a total impairment score by summing the reported compulsion and obsession impairment scores. In the current study, total symptom scores were used as predictors and they had good internal consistency (α = .83).

#### ADHD Symptoms.

The Strengths and Weakness of Attention-Deficit/Hyperactivity Disorder Symptoms and Normal Behavior Scale (SWAN; Swanson et al., 2012) was used to measure ADHD with two subscales, including inattention and hyperactivity/impulsivity. The SWAN is an 18-item parent-report questionnaire that uses a 7-point Likert scale to gauge children’s behavior compared to their peers. Items were rated from −3 (“Far Above Average”) to 0 (“Average”) and then to 3 (“Far Below Average”). higher scores indicate greater levels of behavioral difficulty, while lower scores indicate behavioral strength. Subscale scores are calculated as the mean of items. In the current study, both subscales were assessed as separate predictors, which demonstrated strong internal consistency (α = .89 and α = .92, respectively).

#### Subclinical-Autism Traits.

The Social Responsiveness Scale (SRS) was used to measure the presence and severity of autistic traits in natural social settings. It is a 65-item parent-report of autism traits across five domains: social awareness, social cognition, social communication, social motivation, and restricted interests/repetitive behaviors (Constantino et al., 2003). Items were rated from 0 (“Never True”) to 3 (“Almost Always True”) and converted to T-scores that are standardized to normative groups (α = .92). While the current study excluded children who met diagnostic criteria for ASD, we included this scale to examine subclinical autistic traits, as research has shown that autistic traits are distributed continuously on the spectrum in the general population rather than as a categorical diagnosis (Constantino & Todd, 2003; Marinopoulou, Billstedt, Wessman, Bornehag, & Hallerbäck, 2023). Investigating these subclinical symptoms allows us to examine these milder yet potentially impactful autism-related presentations. Follow-up analyses examined the two empirically derived DSM-5 domain factors of social communication/interaction and restricted/repetitive behavior, detailed in Frazier et al. (2014).

#### Sensory Processing.

Two measures of sensory processing were included to capture both sensory over-responsivity and sensory-seeking behaviors. First, the Sensory Over-Responsivity Scale (SensOR; Schoen et al., 2008) was measured to assess sensory over-responsivity across multiple modalities by identifying things/situations that bother the child. In the current study, 47 yes/no SensOR items in garments (7 items), self-care (7 items), tactile (4 items), visual (4 items), smells (4 items), food/eating (4 items), sounds (4 items), places (6 items), and movement (7 items) domains were administered to the parent. Items from the food and eating subscale were removed to avoid overlap with the primary outcome. All items were rated 0 or 1 and a composite sum score of the remaining 43 items was used in analyses (SensOR without food/eating items; α = .84). Second, the Short Sensory Profile (SSP; McIntosh et al., 1999) was administered to assess sensory processing patterns in children. It is a 38-item parent-report questionnaire with items rated from 1 (“Always”) to 5 (“Never”) and seven subscales. The under-responsive/seeks sensation subscale was used in the current study and is calculated as the sum of seven items (α = .79).

#### Disgust Sensitivity.

The Child Disgust Scale (CDS; Viar-Paxton et al., 2015) was administered to children to assess disgust sensitivity. It is a 14-item questionnaire with items rated from 0 (“Always”) to 2 (“Never”) and two subscales, including disgust affect and disgust avoidance. Both subscales were calculated as the mean of items and used as separate predictors (disgust affect (α = .58), disgust avoidance (α = .73)).

#### Overcontrolled Temperament.

The Overcontrol in Youth Checklist (Gilbert et al., 2020a) is an 18-item parent-report measure developed to assess overcontrolled personality traits in young children, including inhibitory control, rigidity, perfectionism, and excessive need for control. Items were rated with 0 (“No”) or 1 (“Yes”) and computed the total scores as the sum of all items. In the current study, total scores were used as a predictor (α = .85).

#### Socioeconomic Status.

Socioeconomic status (SES) was measured using the income-to-needs ratio, which is calculated by dividing the family’s household income by the federal poverty threshold for the corresponding year of data collection and household size. This was included as an additional predictor given prior studies relating picky eating to lower SES, and the relationship between SES and mental health symptoms more broadly (Cardona et al., 2015; Peverill et al., 2021).

### 2.3. Analysis

Pearson’s correlations were computed between NIAS-PE and demographic variables, including age, sex, race, Hispanic ethnicity, BMI, and income-to-needs ratio, to assess potential confounders. No demographic variables had a statistically significant correlation with NIAS-PE score, but because of the commonly known relationship between picky eating becoming non-normative and maladaptive during this developmental age range, child age was included as a covariate in each of the statistical models.

Multiple imputation methods were utilized to account for missing data. The MI and MIANALYZE procedures in SAS v9.4 (SAS Institute Inc., Cary, NC, USA, 2023) were used to create 25 imputed datasets and pool results from analyses conducted in each of the datasets. Specifically, NIAS outcomes, the 12 candidate predictors, individual subscales of the SRS, and the covariate age were included in the multiple imputation process to ensure no missing data for any variables used in the planned analyses. Parameter estimates and standard errors generated by conducting the regression models described below in each of the 25 datasets were pooled to generate final model results.

We first conducted 12 separate linear regression models, each including one candidate predictor of concurrent picky eating (NIAS-PE score), covarying for age. Next, all predictors that were significant at *p* < .05 (after accounting for multiple comparisons with the False Discovery Rate (FDR)) were then entered into one multivariable linear regression model with NIAS-PE scores as the dependent variable, controlling for age, to identify independent predictors of picky eating severity. As a supplemental analysis, we also examined the other subscales of the NIAS, including the fear of aversive consequences subscale (NIAS-F) and appetite/lack of interest subscale (NIAS-A) using the same approach. Results pertaining to NIAS-F and NIAS-A are reported in Supplemental Tables 1–4.

To follow up on findings that autism-related traits (SRS score) were significantly associated with picky eating severity in the multivariable model, secondary analyses examined the independent contributions of each subscale of the total SRS (social awareness, social cognition, social communication, social motivation, and restricted interests/repetitive behaviors) to NIAS-PE score in separate general linear regression models that covaried for age. Then, to further examine the relationship between domains of autism traits on picky eating severity, each of the subscales significant after FDR correction were re-entered into a separate, multivariable linear regression model. Finally, this same approach was applied to the two empirically derived SRS DSM-5 domain factors (social communication/interaction and restricted/repetitive behavior), first examining the factors separately and then combined in a single linear regression model covarying for age.

All statistical analyses were conducted using SAS version 9.4 software (SAS Institute Inc., Cary, NC, USA, 2023).

## 3. Results

### 3.1. Sample characteristics

The sample consisted of 323 children aged 4 to 7 years (M [SD] = 5.96 [1.10] years) and was 51.1% female (N = 165). The majority of the sample identified as White (76.5%, N = 247). Clinically, 18.3% met criteria for at least one anxiety disorder, 16.2% of participants met criteria for Generalized Anxiety Disorder, and fewer children met criteria for Separation Anxiety or Social Anxiety (both 2.8%). Additional demographic and diagnostic information is found in Table 1.

### 3.2. Preliminary analyses

Our sample had NIAS-PE scores across the full possible range (0–15), with a mean of 5.79 (SD = 4.52) and 73 (23.0%) participants scoring above the clinical cutoff of nine. No demographic variable including age (*r* = −0.035, *p* = .533), female sex (*r* = −0.075, *p* = .184), BMI (*r* = −0.084, *p* = .141), White race (*r* = −0.077, *p* = .175), Hispanic ethnicity (*r* = 0.046, *p* = .419), or income-to-needs ratio (*r* = −0.035, *p* = .541) was significantly associated with NIAS-PE score. Correlations between clinical symptoms, psychological factors, and picky eating are shown in Table 2. VIFs were calculated within each multiply imputed dataset and averaged across datasets for candidate predictors and NIAS-PE without multicollinearity concerns (VIFs 1.07 – 2.39). For the five SRS subscales, mean VIF ranged from 1.68 to 3.31.

### 3.3. Associations with picky eating severity

Results of the 12 separate linear regression models are shown in Table 3. Age was included as a covariate in all models, but age was not significantly associated with picky eating severity (NIAS-PE) in any of the models. Controlling for age, six candidate predictors were significantly associated with picky eating severity. These included depression symptoms (RCADS depression T-score; β = 0.174, *t* = 2.98, FDR *p* = .007), obsessive-compulsive symptoms (ChOCI-R-P total; β = 0.183, *t* = 3.27, FDR *p* = .003), autism traits (SRS total T-score; β = 0.296, *t* = 5.37, FDR *p* < .001), sensory over-responsivity (SensOR without food/eating items; β = 0.270, *t* = 4.96, FDR *p* < .001), and overcontrolled personality traits (OCYC total; β = 0.193, *t* = 3.47, FDR *p* = .002). ADHD inattention symptoms were positively associated with picky eating severity, but the finding did not withstand correction for multiple comparisons (SWAN inattention; β = 0.121, *t* = 2.07, FDR *p* = .077). Anxiety symptoms (RCADS anxiety T-score), ADHD hyperactive/impulsive symptoms, sensory under-responsivity, disgust affect, disgust avoidance, and income-to-needs ratio were not significantly associated with picky eating symptoms.

### 3.4. Multivariable models identifying independent predictors of picky eating severity

Results from the multivariable model can be found in Table 4. Once the five predictors that were significant in the separate models were entered into a single, multivariable linear regression model, again controlling for age, only the total SRS T-score (β = 0.198, *t* = 2.74, *p* = .006) and SensOR without food/eating (β = 0.134, *t* = 1.99, *p* = .047) were statistically significant independent predictors of picky eating severity. Depression symptoms (β = −0.027, *t* = −0.37, *p* = .711), OCD symptoms (β = 0.051, *t* = 0.79, *p* = .431), and overcontrolled personality traits (β = 0.050, *t* = 0.77, *p* = .443) were all non-significant when modeled simultaneously, consistent with overlapping variance among these constructs and the two variables that remained significant (subclinical autistic traits and sensory over-responsivity).

### 3.5. Secondary analyses

In separate general linear regression models of NIAS-PE controlling for age, all five SRS subscales were significantly associated with picky eating severity after FDR correction. Associations were significant for subscales: SRS social awareness (β = 0.174, *t* = 3.07, FDR *p* = .002), SRS social cognition (β = 0.247, *t* = 4.44, FDR *p* < .001), SRS social communication (β = 0.249, *t* = 4.45, FDR *p* < .001), SRS social motivation (β = 0.271, *t* = 4.88, FDR *p* < .001), and SRS restricted interests/repetitive behaviors (β = 0.262, *t* = 4.53, FDR *p* < .001). When entering these significant predictors into a multivariable model including age as a covariate (see Table 5), social motivation was the only statistically significant independent predictor (β = 0.163, *t* = 2.29, *p* = .022). Given that SRS subscales do not fully align with the empirically derived factors of the SRS (Frazier et al., 2014) that also reflect the two DSM-5 categories of ASD diagnosis, follow-up analyses used an empirically derived two-factor structure of the SRS which includes social communication/interaction and restricted/repetitive behavior. Findings paralleled the SRS subscale findings such that only difficulties in social communication/interaction showed significant relationships with picky eating when both factors were entered simultaneously (Supplemental Table 5).

We also examined the two other NIAS subscales, fear of aversive consequences (NIAS-F) and appetite/lack of interest (NIAS-A), using a parallel analytic approach (Supplemental Table 1–4). Although multiple associations between candidate predictors and NIAS-F and NIAS-A emerged, no candidate predictor remained independently significant when modeled simultaneously for either subscale, though the covariate of age was associated with lower NIAS-A scores (β = −0.122, *t* = −2.25, *p* = .025).

## 4. Discussion

The results of the current study replicate prior work showing associations between picky eating and a wide variety of psychiatric clinical symptoms and psychological factors in community children aged 4 – 7 years old. Importantly though, when candidate concurrent predictors were modeled together, subclinical autistic traits (specifically deficits in social motivation) and sensory over-responsivity emerged as the only independent correlates to picky eating. This is notable given that multiple constructs, including depression, obsessive-compulsive symptoms and overcontrolled temperament were significant in individual regression models. It suggests that, relative to the other constructs examined, subclinical autistic traits (social motivation deficits in particular) and sensory overresponsivity may be uniquely related to picky eating as it is first becoming non-normative.

Very high rates of picky eating have been found in autistic children, with prevalence estimates between 51 and 89% (Marzulli et al., 2020). Prior work has linked picky eating in autistic individuals to oral texture sensitivity specifically (Rodrigues et al., 2023). Our study, in contrast, used composite measures of sensory processing from which the oral and food related sensory items were removed to avoid redundancy with the primary outcome. Non-food sensory over-responsivity was nonetheless independently associated with picky eating severity, suggesting picky eating may be linked to a broader sensory reactivity phenotype rather than food or oral sensitivities alone. Though there is a well-established relationship between autistic traits and sensory concerns (Kirby et al., 2022), there were no major multicollinearity concerns in our sample, so it is possible that subclinical autistic traits related to social motivation and sensory processing differences represent partially distinct pathways to picky eating severity, with autistic traits accounting for more variance in this sample.

It is notable given that overcontrolled temperament, which this sample was oversampled for, was not an independent correlate of picky eating severity. Overcontrol has been associated with anorexia nervosa (Isaksson et al., 2021) and is theoretically relevant to ARFID given features of heightened rigidity and need for sameness. However, the results of the study suggest that the sensory sensitivity presentation of ARFID is less likely to be directly related to overcontrol, as the variance was better captured by autistic traits, specifically social motivation. Instead, when subclinical autistic traits are accounted for, multiple constructs previously associated with picky eating in the literature and in individual linear regression models, including depression symptoms, obsessive-compulsive symptoms, and overcontrolled temperament were no longer significant. This is consistent with overlapping variance among these constructs and the two variables that remained significant: subclinical autistic traits, and to a lesser extent, sensory over-responsivity. One interpretation is that symptoms of depression, OCD, and overcontrolled temperament may relate to picky eating partly through shared associations with autistic traits and sensory processing differences. This would be consistent with the well-documented overlap between autistic traits and other psychiatric comorbidity (Hossain et al., 2020). However, the cross-sectional design of the study does not allow for causal inference, or rule out other upstream factors not measured (for example, shared method variance such as parent reporting style) that may influence multiple variables.

Among the SRS subscales, social motivation is the primary independent correlate of picky eating severity, which may be explained by the inherently social processes by which young children expand their diets. By preschool, adult and peer modeling is a strong driver of food acceptance, and children more readily adopt foods when they observe parents, teachers, and peers eating them (Addessi et al., 2005; Birch, 1980; Birch & Fisher, 1998; Hendy & Raudenbush, 2000). The effect of modeling is strongest when children feel similar to or want to affiliate with the model (Cruwys et al., 2015). A child with lower social motivation may therefore be less inclined to attend to or to imitate the eating behavior of those around them, potentially depriving them of a primary mechanism by which dietary variety expands at this age. This is particularly salient at the sample age range of this study, ages 4–7, when peer modeling effects are strong and the first ages at which picky eating is non-normative (Cardona et al., 2015). In our sample, age was not significantly associated with picky eating severity, further suggesting that the study population is within the early developmental range of non-normative picky eating. Though subclinical autistic traits are both stable and heritable (Marrus et al., 2020; Robinson et al., 2011), it is also possible that picky eating leads to reductions in social motivation. For example, children who are picky eaters may appear less socially motivated to participate in the large portion of communal gatherings that involve shared meals or snacks.

The findings of this study provide important insight into a possible early developmental pathway to ARFID. The NIAS-PE assesses the sensory sensitivity presentation of ARFID which is closely related to picky eating in that it is characterized by avoidance of food based on sensory qualities and thus limited dietary variety (Zickgraf & Ellis, 2018). Children with reduced social motivation may have a specific vulnerability that prevents them from benefiting from the social modeling that drives dietary expansion, placing them at risk for persistent picky eating, and if psychosocial impairment or medical complications result, an ARFID diagnosis may follow.

The study has several strengths. Simultaneously modeling the 12 candidate predictors allowed direct comparison of relative contributions. The exclusion of participants with ASD while retaining a dimensional measure of autistic traits via the SRS is a methodological strength that allows relevance to a larger portion of the population than those with a clinical ASD diagnosis. Limitations of the study, as mentioned, include the cross-sectional design, which limits causal inference. The SRS-2, used to measure autistic traits, has good sensitivity but lower specificity (Cholemkery et al., 2013, 2014). In addition, the subscales of the SRS-2 (including social motivation) are theoretically derived, though follow-up analyses using the empirically-derived two factor model in Frazier et al. (2014) continue to support the role of socially-related autistic traits over restricted interests and repetitive behaviors in picky eating.

The sample was predominantly white and relatively high income, limiting generalizability to more racially and socioeconomically diverse populations, especially given the influence of culture on eating patterns (Albardan & Platat, 2025). Additionally, because children with ASD were excluded and the current sample was recruited for elevated overcontrolled (rigid, perfectionistic, need-for-control) tendencies, the generalizability of these findings to populations that meet criteria for autism and to broader community samples remains unknown. Finally, with any questionnaire-based measures, there is always a potential for reporting bias.

The findings have several clinical implications, though cautious interpretation is warranted given the cross-sectional design and absence of formal ARFID diagnostic assessment. Unlike other psychiatric symptoms in this sample (e.g., depression, OCD), autistic traits—particularly social motivation deficits—and sensory overresponsivity remained independent correlates to picky eating severity in this age range. Clinicians evaluating severe or persistent picky eating in children that do not meet full criteria for autism should still consider the role of subclinical autistic traits in the child’s presentation, rather than attributing the presentation solely to other comorbid conditions (such as anxiety or OCD).

Clinically, some degree of picky eating in very young children is considered normative, but when picky eating is severe enough to cause medical or psychosocial complications, it meets criteria for an ARFID diagnosis. There are no FDA-approved medications for ARFID, and although large randomized controlled trials of behavioral therapies for ARFID are lacking, emerging therapies include some component of food exposure, psychoeducation, anxiety management, and family involvement (Allam & Attia, 2025). When an obvious comorbidity is driving the ARFID presentation, then the evidence-based treatment should be chosen to address that comorbidity. Our findings, however, may offer additional guidance for ambiguous cases in which no single driving comorbid diagnosis is apparent. In such cases, it may be prudent to select feeding interventions based on external incentives rather than social motivation, to target sensory overresponsivity potentially via multidisciplinary collaboration, and to avoid assuming that patient-therapist relationship will function in the same way as it would for children without elevated subclinical autistic traits, as existing evidence on the capacity for strong psychotherapeutic relationships within autism populations is mixed (Brewe et al., 2021; Kerns et al., 2018).

Future directions include longitudinally following children to track natural trajectories of picky eating, with a full diagnostic assessment of ARFID. Overall, in a community sample enriched for overcontrolled temperament, subclinical autistic traits– specifically deficits in social motivation– emerged as the primary independent correlate of picky eating severity when multiple candidate psychological constructs were modeled together. These findings highlight the role of subclinical autistic traits (particularly social motivation deficits) and sensory processing differences in early non-normative picky eating, with potential implications for ARFID risk stratification and treatment development.

## Supplementary Material

Appendix A

## Figures and Tables

**Table 1:** Subject characteristics.

Demographics	N	Mean	SD	Range
Age	323	5.96	1.10	4.09 – 7.99
Income-to-needs ratio	316	4.51	1.92	0.31 – 10.92
	N	%	n	
Female sex	323	51.1	165	
Hispanic ethnicity	323	7.1	23	
Race	323			
White		76.5	247	
Black		8.7	28	
Asian		1.6	5	
More than one race		12.1	39	
Unknown		1.2	4	
Informant	322			
Biological mother		90.4	291	
Biological father		5.0	16	
Adoptive/epigenetic mother		3.1	10	
Adoptive father		0.6	2	
Other		0.9	3	
Mother’s education	323			
High school		1.9	6	
Some college		5.3	17	
Bachelor’s degree		22.9	74	
Master’s degree		23.2	75	
Graduate degree		8.0	26	
Unknown		38.7	125	
Predictors	N	Mean	SD	Range
RCADS depression T-score	286	48.69	7.92	37.34 – 78.29
RCADS anxiety T-score	286	46.75	8.34	32.10 – 75.47
ChOCI-R-P total	306	1.76	3.04	0 – 32
SWAN inattention	300	−0.39	0.82	−2.88 – 1.89
SWAN hyperactive/impulsive	300	−0.17	0.86	−2.78 – 2.11
SRS total T-score	300	46.04	6.42	35 – 67
SensOR (w/o food/eating)	318	5.94	5.09	0 – 29
SSP underresponsive	317	25.24	4.66	9 – 35
CDS affect	311	0.64	0.50	0 – 2
CDS avoidance	312	1.54	0.40	0 – 2
OCYC total	323	8.18	4.59	0 – 18
Outcomes	N	Mean	SD	Range
NIAS picky eating	318	5.79	4.52	0 – 15
NIAS fear	318	0.74	1.89	0 – 12
NIAS appetite	318	3.83	4.00	0 – 15
Current Diagnoses	N	%	n	
Anxiety disorder	322	18.3	59	
Generalized anxiety disorder	321	16.2	52	
Social anxiety disorder	322	2.8	9	
Separation anxiety disorder	322	2.8	9	
Obsessive-compulsive disorder	322	1.9	6	
Major depressive disorder	321	0.6	2	
Externalizing disorder	319	4.4	14	
Attention-deficit/hyperactivity disorder	319	3.5	11	
Oppositional defiant disorder	315	1.3	4	
Conduct disorder	313	0.0	0	
Post-traumatic stress disorder	320	0.3	1	

*Note*: RCADS = The Revised Children’s Anxiety and Depression Scale - Parent Version, ChOCI-R-P = The parent version of the Revised Children’s Obsessional Compulsive Inventory, SWAN = The Strengths and Weakness of Attention-Deficit/Hyperactivity Disorder Symptoms and Normal Behavior Scale, SRS = The Social Responsiveness Scale, SensOR = The Sensory Over-Responsivity Scale, SSP = The Short Sensory Profile, CDS = The Child Disgust Scale, OCYC = The Overcontrol in Youth Checklist, NIAS = The Nine Item ARFID Screen.

**Table 2:** Correlation matrix of clinical and psychological risk factors and picky eating.

	1	2	3	4	5	6	7	8	9	10	11	12	13	14	15
1. NIAS picky eating	1.00														
2. NIA fear	0.28*	1.00													
3. NIAS appetite	0.59*	0.40*	1.00												
4. RCADS depression	0.16^	0.18^	0.25*	1.00											
5. RCADS anxiety	0.08	0.11	0.06	0.66*	1.00										
6. ChOCI-R-P total	0.16^	0.22^	0.19^	0.46*	0.52*	1.00									
7. SWAN inattention	0.11	−0.06	0.13°	0.31*	0.11	0.01	1.00								
8. SWAN hyperact/impulse	0.07	−0.08	0.06	0.38*	0.21^	0.14°	0.72*	1.00							
9. SRS total	0.28*	0.22^	0.30*	0.53*	0.34*	0.39*	0.42*	0.44*	1.00						
10. SensOR w/o food/eating	0.26*	0.15^	0.26*	0.38*	0.40*	0.32*	0.15°	0.15^	0.55*	1.00					
11. SSP underresponsive	−0.09	−0.09	−0.21^	−0.33*	−0.18^	−0.24*	−0.35*	−0.41*	−0.43*	−0.25*	1.00				
12. CDS affect	0.01	0.03	0.06	−0.00	−0.01	0.10	0.02	0.01	−0.09	−0.01	−0.05	1.00			
13. CDS avoidance	−0.04	0.01	−0.07	−0.01	0.09	0.03	0.02	0.06	0.08	0.10	0.04	−0.28*	1.00		
14. OCYC total	0.18^	0.19^	0.23*	0.48*	0.49*	0.41*	0.02	0.14°	0.38*	0.43*	−0.23*	0.09	−0.03	1.00	
15. Income-to-needs ratio	−0.03	−0.02	−0.06	−0.05	−0.07	−0.13°	0.00	0.01	−0.12°	−0.12°	0.04	0.04	−0.10	0.05	1.00

*Note*: p <.0001,

^ p <.01,

° p <.05.

NIAS = The Nine Item ARFID Screen, RCADS = The Revised Children’s Anxiety and Depression Scale - Parent Version, ChOCI-R-P = The parent version of the Revised Children’s Obsessional Compulsive Inventory, SWAN = The Strengths and Weakness of Attention-Deficit/Hyperactivity Disorder Symptoms and Normal Behavior Scale, SRS = The Social Responsiveness Scale, SensOR = The Sensory Over-Responsivity Scale, SSP = The Short Sensory Profile, CDS = The Child Disgust Scale, OCYC = The Overcontrol in Youth Checklist, NIAS = The Nine Item ARFID Screen.

**Table 3:** Potential predictors of NIAS picky eating in separate general linear regression models covarying for age (N = 323).

Predictor	Est.	SE	*β*	*t*	*p*	FDR *p*
RCADS depression T-score	0.099	0.033	0.174	2.98	0.003	0.007
RCADS anxiety T-score	0.057	0.031	0.108	1.85	0.064	0.110
ChOCI-R-P total	0.272	0.083	0.183	3.27	0.001	0.003
SWAN inattention	0.661	0.319	0.121	2.07	0.038	0.077
SWAN hyperactive/impulsive	0.356	0.301	0.069	1.18	0.237	0.317
SRS total T-score	0.209	0.039	0.296	5.37	<0.001	<0.001
SensOR (w/o food/eating)	0.238	0.048	0.270	4.96	<0.001	<0.001
SSP underresponsive	−0.087	0.054	−0.090	−1.59	0.111	0.167
CDS affect	0.199	0.524	0.022	0.38	0.705	0.705
CDS avoidance	−0.398	0.656	−0.035	−0.61	0.544	0.593
OCYC total	0.190	0.055	0.193	3.47	<0.001	0.002
Income-to-needs ratio	−0.095	0.136	−0.040	−0.69	0.488	0.586

*Note*: RCADS = The Revised Children’s Anxiety and Depression Scale - Parent Version, ChOCI-R-P = The parent version of the Revised Children’s Obsessional Compulsive Inventory, SWAN = The Strengths and Weakness of Attention-Deficit/Hyperactivity Disorder Symptoms and Normal Behavior Scale, SRS = The Social Responsiveness Scale, SensOR = The Sensory Over-Responsivity Scale, SSP = The Short Sensory Profile, CDS = The Child Disgust Scale, OCYC = The Overcontrol in Youth Checklist.

**Table 4:** General linear regression model of NIAS picky eating including significant predictors from separate models covarying for age (N = 323).

	Est.	SE	*β*	*t*	*p*
Intercept	0.194	2.651	–	0.07	0.942
Age	−0.224	0.223	−0.055	−1.01	0.315
RCADS depression T-score	−0.015	0.041	−0.027	−0.37	0.711
ChOCI-R-P total	0.075	0.096	0.051	0.79	0.431
SRS total T-score	0.139	0.051	0.198	2.74	0.006
SensOR (w/o food/eating)	0.118	0.059	0.134	1.99	0.047
OCYC total	0.049	0.064	0.050	0.77	0.443

*Note*: RCADS = The Revised Children’s Anxiety and Depression Scale - Parent Version, ChOCI-R-P = The parent version of the Revised Children’s Obsessional Compulsive Inventory, SRS = The Social Responsiveness Scale, SensOR = The Sensory Over-Responsivity Scale, OCYC = The Overcontrol in Youth Checklist.

**Table 5:** General linear regression model of NIAS picky eating by social responsiveness scale subscales covarying for age (N = 323).

	Est.	SE	*β*	*t*	*p*
Intercept	−4.274	2.438	–	−1.75	0.080
Age	−0.159	0.224	−0.039	−0.71	0.478
SRS awareness	0.002	0.044	0.003	0.04	0.964
SRS social cognition	0.055	0.058	0.085	0.95	0.342
SRS social communication	0.007	0.073	0.010	0.10	0.923
SRS social motivation	0.093	0.041	0.163	2.29	0.022
SRS restricted interests/repetitive behaviors	0.077	0.055	0.114	1.40	0.163

*Note*: SRS = The Social Responsiveness Scale.

## Data Availability

Data will be made available on request.

## Appendix A. Supplementary data

Supplementary data to this article can be found online at https://doi.org/10.1016/j.appde.2026.100010.
