# Supplementary material for: Social motivation and sensory over-responsivity: Independent correlates of picky eating severity in early childhood
Source: Appet Disord Eat. Author manuscript; Available in PMC 2026 Oct 28. (PMC13697864; doi:10.1016/j.appde.2026.100010)
Supplement: Supplementary file 1 [file NIHMS2216499-supplement-Appendix_A.docx]

**Supplemental Table 1. Potential Predictors of NIAS Fear of Aversive Consequences (NIAS-F) in Separate General Linear Regression Models Covarying for Age (N=323)**

| **Predictor** | **Est.** | **SE** | **β** | **t** | **p** | **FDR p** |
| --- | --- | --- | --- | --- | --- | --- |
| RCADS depression T-score | 0.054 | 0.015 | 0.225 | 3.56 | <0.001 | 0.001 |
| RCADS anxiety T-score | 0.046 | 0.013 | 0.210 | 3.52 | <0.001 | 0.001 |
| ChOCI-R-P total | 0.136 | 0.035 | 0.218 | 3.85 | <0.001 | <0.001 |
| SWAN inattention | -0.147 | 0.139 | -0.064 | -1.06 | 0.290 | 0.387 |
| SWAN hyperactive/impulsive | -0.191 | 0.129 | -0.088 | -1.48 | 0.139 | 0.209 |
| SRS total T-score | 0.067 | 0.017 | 0.229 | 3.95 | <0.001 | <0.001 |
| SensOR (w/o food/eating) | 0.058 | 0.020 | 0.158 | 2.84 | 0.005 | 0.009 |
| SSP underresponsive | -0.039 | 0.023 | -0.096 | -1.71 | 0.088 | 0.150 |
| CDS affect | 0.042 | 0.218 | 0.011 | 0.19 | 0.846 | 0.846 |
| CDS avoidance | 0.085 | 0.272 | 0.018 | 0.31 | 0.755 | 0.824 |
| OCYC total | 0.079 | 0.023 | 0.193 | 3.47 | <0.001 | 0.001 |
| Income-to-needs ratio | -0.020 | 0.059 | -0.021 | -0.35 | 0.729 | 0.824 |

*Note:* RCADS= R

**Supplemental Table 2. General Linear Regression Model of NIAS Fear of Aversive Consequences (NIAS-F) Including Significant Predictors from Separate Models Covarying for Age (N=323)**

|  | **Est.** | **SE** | **β** | **t** | **p** |
| --- | --- | --- | --- | --- | --- |
| Intercept | -2.505 | 1.249 | -- | -2.01 | 0.045 |
| Age | 0.004 | 0.095 | 0.002 | 0.04 | 0.969 |
| RCADS depression T-score | 0.014 | 0.024 | 0.061 | 0.60 | 0.548 |
| RCADS anxiety T-score | 0.011 | 0.020 | 0.052 | 0.56 | 0.578 |
| ChOCI-R-P total | 0.055 | 0.045 | 0.089 | 1.21 | 0.225 |
| SRS total T-score | 0.037 | 0.024 | 0.126 | 1.54 | 0.123 |
| SensOR (w/o food/eating) | -0.003 | 0.026 | -0.008 | -0.12 | 0.907 |
| OCYC total | 0.025 | 0.028 | 0.060 | 0.89 | 0.374 |

**Supplemental Table 3. Potential Predictors of NIAS Appetite/Lack of Interest (NIAS-A) in Separate General Linear Regression Models Covarying for Age (N=323)**

| **Predictor** | **Est.** | **SE** | **β** | **t** | **p** | **FDR p** |
| --- | --- | --- | --- | --- | --- | --- |
| RCADS depression T-score | 0.139 | 0.029 | 0.276 | 4.76 | <0.001 | <0.001 |
| RCADS anxiety T-score | 0.052 | 0.028 | 0.112 | 1.90 | 0.058 | 0.087 |
| ChOCI-R-P total | 0.288 | 0.073 | 0.219 | 3.93 | <0.001 | <0.001 |
| SWAN inattention | 0.654 | 0.279 | 0.135 | 2.35 | 0.019 | 0.032 |
| SWAN hyperactive/impulsive | 0.275 | 0.264 | 0.060 | 1.04 | 0.298 | 0.325 |
| SRS total T-score | 0.191 | 0.034 | 0.308 | 5.62 | <0.001 | <0.001 |
| SensOR (w/o food/eating) | 0.211 | 0.042 | 0.271 | 5.00 | <0.001 | <0.001 |
| SSP underresponsive | -0.180 | 0.047 | -0.210 | -3.83 | <0.001 | <0.001 |
| CDS affect | 0.530 | 0.467 | 0.066 | 1.14 | 0.256 | 0.325 |
| CDS avoidance | -0.554 | 0.579 | -0.056 | -0.96 | 0.339 | 0.339 |
| OCYC total | 0.221 | 0.048 | 0.254 | 4.65 | <0.001 | <0.001 |
| Income-to-needs ratio | -0.126 | 0.120 | -0.061 | -1.05 | 0.293 | 0.325 |

**Supplemental Table 4. General Linear Regression Model of NIAS Appetite/Lack of Interest (NIAS-A) Including Significant Predictors from Separate Models Covarying for Age (N=323)**

|  | **Est.** | **SE** | **β** | **t** | **p** |
| --- | --- | --- | --- | --- | --- |
| Intercept | 1.084 | 3.194 | -- | 0.34 | 0.734 |
| Age | -0.443 | 0.197 | -0.122 | -2.25 | 0.025 |
| RCADS depression T-score | 0.043 | 0.038 | 0.086 | 1.15 | 0.253 |
| ChOCI-R-P total | 0.060 | 0.087 | 0.046 | 0.69 | 0.491 |
| SWAN inattention | 0.126 | 0.313 | 0.026 | 0.40 | 0.688 |
| SRS total T-score | 0.074 | 0.050 | 0.119 | 1.49 | 0.137 |
| SensOR (w/o food/eating) | 0.078 | 0.052 | 0.100 | 1.50 | 0.133 |
| SSP underresponsive | -0.052 | 0.053 | -0.061 | -0.99 | 0.324 |
| OCYC total | 0.080 | 0.057 | 0.092 | 1.41 | 0.160 |

**Supplemental Table 5.** **General Linear Regression Models of NIAS Picky Eating by Social Responsiveness Scale Empirically Derived Factors (Separately and Together) Covarying for Age (N=323)**

| **SRS Social Communication/Interaction Factor** | **Est.** | **SE** | **β** | **t** | **p** |
| --- | --- | --- | --- | --- | --- |
| Intercept | 4.224 | 1.413 | -- | 2.99 | 0.003 |
| Age | -0.182 | 0.221 | -0.044 | -0.82 | 0.412 |
| SRS social communication/interaction | 0.123 | 0.023 | 0.289 | 5.23 | <0.001 |
| **SRS Restricted/Repetitive Behavior Factor** | **Est.** | **SE** | **β** | **t** | **p** |
| Intercept | 5.039 | 1.396 | -- | 3.61 | <0.001 |
| Age | -0.145 | 0.223 | -0.035 | -0.65 | 0.516 |
| SRS restricted/repetitive behavior | 0.160 | 0.035 | 0.257 | 4.55 | <0.001 |
| **Both SRS Factors** | **Est.** | **SE** | **β** | **t** | **p** |
| Intercept | 4.194 | 1.411 | -- | 2.97 | 0.003 |
| Age | -0.175 | 0.221 | -0.042 | -0.79 | 0.430 |
| SRS social communication/interaction | 0.090 | 0.034 | 0.213 | 2.67 | 0.008 |
| SRS restricted/repetitive behavior | 0.068 | 0.050 | 0.109 | 1.35 | 0.176 |

**Supplemental Table 6. Social Responsiveness Scale Subscale Correlations (N=323)**

|  | 1 | 2 | 3 | 4 | 5 |
| --- | --- | --- | --- | --- | --- |
| 1. SRS awareness | 1.000 |  |  |  |  |
| 2. SRS social cognition | 0.577 | 1.000 |  |  |  |
| 3. SRS social communication | 0.643 | 0.745 | 1.000 |  |  |
| 4. SRS social motivation | 0.366 | 0.490 | 0.612 | 1.000 |  |
| 5. SRS restricted interests/repetitive behaviors | 0.487 | 0.638 | 0.645 | 0.522 | 1.000 |

All p’s < 0.0001
